# Advancing Non-Invasive Diagnosis of Oral Epithelial Dysplasia: Comparative Insights from In Vivo Optical Coherence Tomography and Histopathology

**DOI:** 10.3390/jcm14041118

**Published:** 2025-02-09

**Authors:** Waseem Jerjes, Zaid Hamdoon, Dara Rashed, Colin Hopper

**Affiliations:** 1 Faculty of Medicine, Imperial College London, London SW7 2AZ, UK; 2 Research and Development Unit, Hammersmith and Fulham PCN, London W6 7HY, UK; 3 Unit of OMFS, UCL Eastman Dental Institute, London WC1E 6DE, UK; zothman@sharjah.ac.ae (Z.H.); d.rashed@alumni.ucl.ac.uk (D.R.); c.hopper@ucl.ac.uk (C.H.); 4 College of Dental Medicine, University of Sharjah, Sharjah P.O. Box 27272, United Arab Emirates

**Keywords:** optical coherence tomography, oral epithelial dysplasia, carcinoma in situ, non-invasive imaging, diagnostic accuracy, oral cancer detection

## Abstract

**Background:** Oral epithelial dysplasia (OED) is considered one of the premalignant lesions for oral squamous cell carcinoma (OSCC), for which the five-year disease-free survival rate may vary widely. There has emerged in recent years, therefore, a significant niche for optical coherence tomography (OCT) to non-invasively examine tissue morphology. The present study was conducted to evaluate the diagnostic performance of OCT in distinguishing between mild, moderate, and severe dysplasias and carcinoma in situ (CIS) with histopathological correlations. **Methods:** This prospective, single-centre study included 120 patients with clinically suspicious oral lesions. All lesions underwent in vivo OCT imaging followed by surgical excision and a histopathological examination. The sensitivity, specificity, and AUC (area under the curve) were calculated as measures of diagnostic accuracy. **Results:** OCT demonstrated high diagnostic performance with sensitivity and specificity above 80% for all grades of dysplasia. The AUC values were highest for moderate dysplasia at 0.91 and mild dysplasia at 0.89. The Bland–Altman analysis revealed a high degree of agreement between OCT and histopathology regarding the tumour depth measurements. Interobserver agreement was substantial to almost perfect, with kappa values ranging from 0.74 to 0.85. OCT provided the key imaging features of epithelial thickening, basement membrane disruption, and architectural disorganization. These had good correlations with the grade of dysplasia: r = 0.75–0.82, *p* < 0.001. **Conclusions:** OCT is an established diagnostic technique that is non-invasive in nature for the diagnosis of OED; it can provide fine differentiation among grades of dysplasia and define the margins of a lesion.

## 1. Introduction

OED represents a spectrum of histopathological changes that range from mild dysplasia to carcinoma in situ. These lesions are considered precancerous and are usually associated with an increased risk of malignant transformation into oral squamous cell carcinoma, a significant global health burden. OSCC is the sixth most common malignancy worldwide, accounting for 2% of all malignancies, with approximately 389,485 new cases and 188,230 deaths annually [1]. The prognosis for patients with OSCC is still poor, who have a 5-year survival rate of around 50%, underlining the importance of an early diagnosis and intervention [2].

The aetiology of OED and OSCC is multicausal, with clearly established risk factors, including the use of tobacco, alcohol consumption, betel quid chewing, and chronic mechanical irritation. More recently, evidence is emerging on the role of human papillomavirus (HPV), particularly HPV-16, in the pathogenesis of some subsets of oral lesions [3]. Further contributing to these diseases are lifestyle factors and socio-economic disparities in incidence and disease outcomes [4].

Clinically, oral lesions may present with subtle and nonspecific features, making a diagnosis challenging. The most common presentations include leucoplakia, erythroplakia, and mixed lesions. Though leucoplakia is more common, erythroplakia and mixed lesions have higher risks of harbouring dysplasia or malignancy. The differential diagnosis of benign vs. malignant potential lesions is particularly difficult in view of the overlap of clinical and histopathological features [5].

Histopathology has remained the gold standard in the diagnosis of OED, where the diagnosis is confirmed by biopsy through an invasive procedure followed by a microscopic review. However, this has several drawbacks: biopsy samples are only a very small part of the lesion and might miss the foci of higher-grade dysplasia. The biopsy procedure carries the risk of patient discomfort, infection, and bleeding complications [6]. Observer variability among pathologists is another challenge in borderline cases [7]. However, the limitations of biopsy, including sampling errors and procedural complications, highlight the need for adjunct diagnostic tools like OCT rather than outright replacement.

The limitations of conventional diagnostic methods have stimulated interest in non-invasive imaging modalities for the real-time assessment of lesions. The most promising techniques yielding encouraging preliminary results in the identification of dysplastic changes include autofluorescence imaging, narrowband imaging, and confocal microscopy. However, these techniques very often fail to provide specific diagnostic results and are not routine in clinical practice due to their cost and complexity [8].

The current development has turned optical coherence tomography into a revolutionary, real-time imaging modality that offers high-resolution, cross-sectional views of the tissue architecture. Utilizing near-infrared light, OCT provides highly detailed information about the stratification of the epithelium, integrity of the basement membrane, and subepithelial structures, thus being very well-suited for mucosal lesion assessments. Since OCT imaging is non-invasive, its ability to image large areas without the removal of tissues makes it a potentially revolutionary diagnostic and management tool in the very early detection of OED [9].

Several research works have used OCT as a method for distinguishing dysplastic or malignant tissue from normal oral mucosa. Epithelial thickening, architectural disorganization, and a loss of basement membrane integrity characterize the status of dysplasia or carcinoma in a suspicious lesion and are observable in OCT images. Reflective patterns in the epithelium and subepithelial stroma further categorize the grades of the dysplasias [10]. Despite such advances, difficulties still persist in the field of standardization of the interpretation of OCT and enabling its use in the routine flow of clinics.

New developments in OCT that include machine learning algorithms for the analysis of images have increased the accuracy of diagnostics. Sensitivity and specificity rates in the detection of dysplasia have been reported in several studies as being above 85%, while the AUC (area under the curve) often surpasses 0.9 for high-grade lesions [11]. However, with variable performance reported across lesion types, anatomical locations, and operator expertise, further validation will be needed in a range of clinical settings.

Interest within the clinical field in this OCT is based on the hope that it could, once and for all, minimize-the need for, at the least, an invasive biopsy to decrease patient morbidity and reduce health care spending to a limited number of occasions selected. To date, and with some few exceptions, the results mainly refer to small, selected cases or specific lesion types and therefore cannot easily be generalized. Large series studies properly evaluating the performance of OCT across a broad spectrum of different dysplasia grades are fewer than large prospective validation studies and are critical for making decisions about the clinical roles this can support in practice.

This study, therefore, investigated the diagnostic performance of OCT in distinguishing between mild, moderate, and severe dysplasia, besides CIS, in oral lesions. A correlation of OCT findings with the histopathological examination results was performed to establish its accuracy, reliability, and usefulness in clinical decision-making.

## 2. Patients and Methods

### 2.1. Study Design and Setting

This is a prospective, single-centre study designed to evaluate the diagnostic performance of in vivo optical coherence tomography in differentiating mild, moderate, and severe dysplasia and carcinoma in situ of oral epithelial lesions. The study was conducted in a tertiary care centre (UCLH Head and Neck Centre) that specializes in oral oncology. Ethical clearance was obtained from the Institutional Review Board (Moorfields and Whittington Local Research Ethics Committee of the National Health Service England, REC reference number 07/Q0504/4, protocol code 1, and date of approval 21 February 2017), and all participants provided written informed consent before inclusion. The study was carried out in accordance with the principles of the Declaration of Helsinki.

### 2.2. Imaging Protocol

In vivo imaging of all lesions was conducted using a high-resolution OCT system, such as the one provided by VivoSight^®^ developed by Michelson Diagnostics (Maidstone, UK). A near-infrared light source with a wavelength of 1305 nm is well suited for acquiring high-resolution cross-sectional and en face images of the oral mucosa. The axial resolution of this system is 5 μm, while the lateral resolution is 7.5 μm, providing nice details on epithelial stratification, basement membrane integrity, and subepithelial structures.

OCT imaging was performed by two trained clinicians who were blinded to the clinical and histopathological results. In each lesion, scanning was performed to cover the lesion and at least 2 mm of surrounding normal tissue to capture information on the epithelial thickness, architectural disorganization, and reflectivity patterns. The scanning time per lesion took about 5–10 min. OCT images were analysed offline by two independent observers with experience in oral mucosal imaging. Systematic documentation of epithelial stratification and disruption of the basement membrane were the key features.

### 2.3. Surgical Excision and Histopathology

Following OCT imaging, all lesions were surgically excised under local anaesthesia by a specialist surgeon. Excision margins were chosen based on the clinical and OCT findings to ensure at least 2 mm beyond the lesion’s clinically and OCT-measured edge. The specimens were immediately fixed with 10% neutral-buffered formalin, oriented, and inked for the margin assessment.

The histopathological assessment was performed independently by two experienced pathologists who were independent of those who examined the OCT results. Their grading of dysplasia, that is, mild dysplasia, moderate, severe, or CIS, came using WHO criteria regarding the specific architectural disorganization, nuclear changes, and mitotically active features. The tumour size and lateral extension were examined and then compared with estimations obtained by OCT testing. Any differences in opinion provided by the pathologists concerned were settled by consensus with a third expert pathologist or opinion.

### 2.4. Statistical Analysis

The primary endpoints of the study were the sensitivity, specificity, PPV, and NPV of OCT in differentiating the grade of dysplasia and CIS. Secondary endpoints included the accuracy of OCT regarding the tumour depth and margin status, and interobserver agreement for the OCT interpretation.

The baseline characteristics of patients and lesions are described descriptively. Diagnostic performance measures were determined along with 95% CIs. Receiver operating characteristic (ROC) curves were obtained for OCT to differentiate between grades of dysplasia. The AUC was used as a measure of diagnostic accuracy. The Bland–Altman analysis was used to assess the agreement between the OCT-derived and histopathology-confirmed tumour depth. Interobserver agreement was evaluated using Cohen’s kappa coefficient.

## 3. Results

### 3.1. Study Population and Patient Characteristics

A total of 120 adult patients presenting with clinically suspicious oral lesions were enrolled in the study. The mean age of participants was 56.8 ± 11.7 years, while the age range was from 29 to 81 years. There was a slight predominance of males, having 67 males (56%) and 53 females (44%). Consecutive patients were recruited from outpatient clinics to ensure a representative cohort of individuals commonly presenting with high-risk oral lesions (Table 1).

The cohort of patients had a high prevalence of known risk factors for OED. The smoking history was significant, with 72 patients (60%) being current or former smokers. Of these, 46 patients (38%) were current smokers, while 26 patients (22%) were former smokers who had quit for a median duration of 5 years (range: 1–20 years). The mean cumulative smoking exposure was 14.8 ± 9.2 pack-years, with heavy smoking (≥20 pack-years) in 18 patients (15%). Forty-eight patients were never smokers (40%).

Sixty-eight patients reported drinking alcohol. Of these, 19 patients reached the criteria for heavy drinking, defined as ≥14 units/week. The mean weekly alcohol intake among drinkers was 18.2 ± 7.5 units, with a significant subset consuming in excess of 30 units per week, 12 patients (10%). Smoking in combination with heavy alcohol use was seen in 22 (18%) patients, and is a well-known synergistic risk factor for oral premalignant lesions.

Among the subjects, 19 (16%) were found to engage in betel quid chewing or areca nut use, while 12 were considered habitual users of this substance (≥3 times per week for more than one year). Meanwhile, 32 patients (27%) had chronic irritation because of ill-fitting dentures, sharp dental restorations, or habitual cheek or tongue biting. Poor oral hygiene—termed as visible plaque, gingiva inflammation, or rare dental check-ups—accounted for 52 patients (43%).

The study cohort did, however, comprise quite a number of medically compromised patients with conditions known to interfere with the normal functioning of the oral epithelium. A medical history of diabetes mellitus was also available for 21 patients with poor mucosal immunity associated with a history of being treated for this disorder that resulted in delayed healing among diabetic patients. Drugs predisposing the patients to develop xerostomia included, amongst others, antihypertensives, antidepressants, and/or anticholinergics in 24 subjects during an investigation into current oral dryness and irritation of the oral mucosa. In 12 patients, a clinical suspicion of human papillomavirus infection, a known risk factor for certain oral dysplastic lesions, was raised based on the lesion morphology and risk factors.

### 3.2. Lesion Characteristics

A total of 120 oral lesions were analysed, all measuring ≤ 10 mm in the maximum dimension. Lesions were distributed across high-risk anatomical sites: the lateral tongue was the most common location, with 42 lesions (35%), followed by the buccal mucosa with 28 lesions (23%), the floor of the mouth with 26 lesions (22%), soft palate with 14 lesions (12%), and the retromolar trigone with 10 lesions (8%). These are known sites of high risk by virtue of being susceptible to more chronic irritation and carcinogen exposure (Table 2).

Clinically, the lesions were divided into leucoplakia, erythroplakia, or mixed lesions. Seventy-eight lesions (65%) presented as leucoplakia, of which 50 lesions presented homogeneously (42%) and 28 lesions presented non-homogeneously (23%). Erythroplakia, which was reported to be highly associated with severe dysplasia or CIS, was found in 28 lesions (23%); 14 (12%) were classified as mixed red and white lesions. These had ulceration or surface irregularity, raising the clinical suspicion of a higher grade of dysplasia in 36 (30%) lesions.

Lesion dimensions were measured with standardized digital callipers. The mean lesion size was 7.2 ± 1.8 mm, ranging between 4 and 10 mm. Surface features such as texture (smooth vs. rough), keratinization, and ulceration were recorded in a standardized fashion clinically to aid in the OCT interpretation and histopathological correlation.

### 3.3. Histopathological Grading of Lesions

The histopathological examination showed that the 120 lesions in this study were distributed as follows: mild dysplasia, 40 cases (33.3%); moderate dysplasia, 36 cases (30.0%); severe dysplasia, 28 cases (23.3%); and CIS, 16 cases (13.3%) (Table 3). This further explains the clinical importance of this cohort, given that about two-thirds of the lesions consisted of higher-grade dysplasia or CIS.

### 3.4. Diagnostic Accuracy of OCT Across Dysplasia Grades

**Figure 1:**
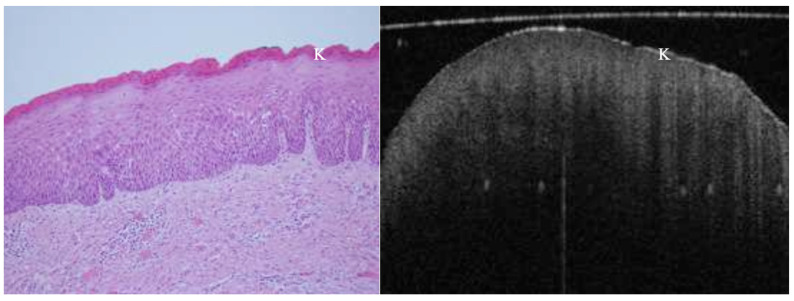
In vivo OCT and histopathology images of a white patch on the posterior lateral tongue reveal keratosis and mild to moderate epithelial dysplasia. K = keratosis.

**Figure 2:**
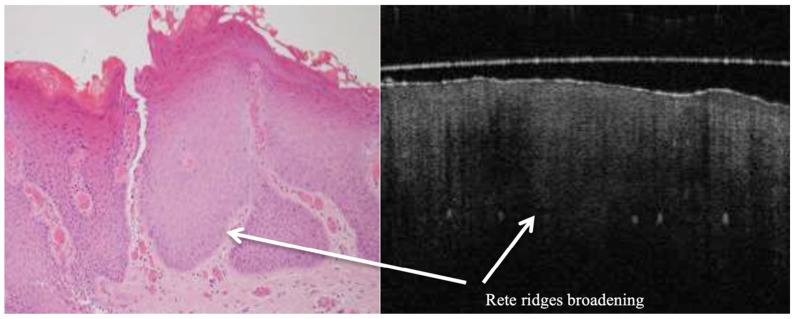
In vivo OCT and histopathology images divulge keratosis and moderate epithelial dysplasia of an erythroplakic lesion on the anterior lateral tongue.

**Figure 3:**
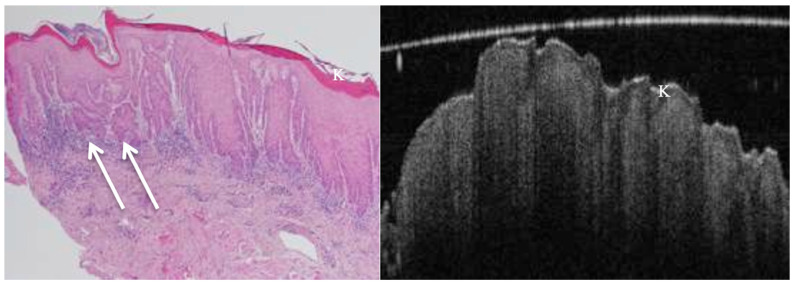
OCT (in vivo) and histopathology images reveal hyperkeratosis and severe epithelial dysplasia of a speckled leucoplakia on floor of mouth. Early invasive carcinoma (arrows) shown in the histology image cannot be visualised on the corresponding OCT image due to lack of sufficient depth of penetration as well as the inferior image resolution compared to the H&E-stained section. K = keratosis.

**Figure 4:**
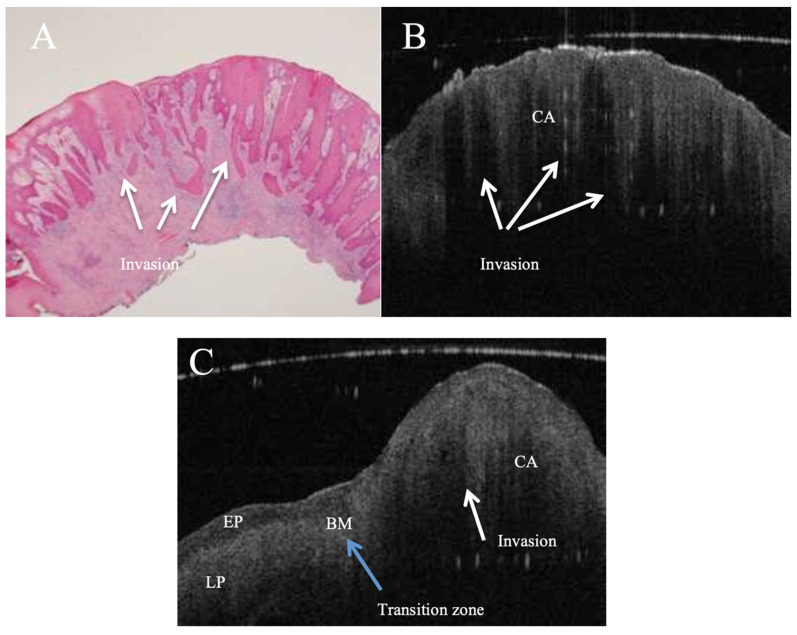
In vivo OCT and histopathology images (**A**,**B**) of an erythro–leukoplakic lesion of the soft palate revealing multifocal carcinoma (CA). The OCT image matches the histopathology in displaying multifocal epithelial downwards growth and invasion into the subepithelial layers (white arrows). Furthermore, the basement membrane is indiscernible through the entire OCT scan as a coherent prominent landmark. The bottom image (**C**) is an in vivo OCT image of a transition zone (blue arrow) between the healthy tissue and invasive carcinoma (CA) invading through the basement membrane. It also shows the normal thickness of the stratified squamous epithelium, which is darker compared to the homogeneous lamina propria; while crossing the transition zone, epithelial downgrowth and invasion can be clearly recognised, and the lamina propria becomes non-homogeneous. BM = basement membrane; EP = epithelium; LP = lamina propria; CA = invasive carcinoma.

OCT performed well in discriminating dysplasia grades (Figure 1, Figure 2, Figure 3 and Figure 4). The AUC was 0.89 with an 88.0% sensitivity and 90.0% specificity for mild dysplasia. For moderate dysplasia, the highest accuracy was found: 85.0% sensitivity and 92.0% specificity yielded an AUC of 0.91. Severe dysplasia and CIS presented with lower values: the AUCs were 0.87 and 0.86, respectively (Table 4).

**Figure 5:**
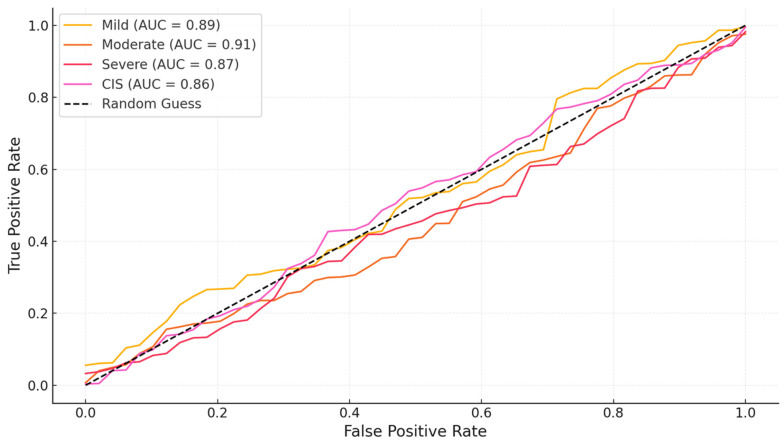
ROC curve for dysplasia grades. This ROC curve shows the diagnostic performance of OCT for each dysplasia grade, with the AUC values indicating accuracy.

In Figure 5, the ROC curve of the OCT diagnostic capability for each dysplasia grade is shown. Figure 5 presents the AUC, confirming the high discriminatory power of OCT, in particular for moderate dysplasia. The steep rise in the ROC curve towards the upper left corner illustrates the accuracy in the separation of true positives from false positives that OCT provides across all lesion grades. It should be noted, however, that the main separation of the curves with regard to different grades emphasizes the capabilities of OCT to differentiate dysplasias of differing severities; minimal overlap occurred between moderate and mild dysplasia.

### 3.5. Agreement Between OCT and Histopathology

OCT predictions of dysplasia grades closely approximated the histopathological diagnoses across the spectrum of dysplasia grades (Table 5). For mild dysplasia, 35 out of 40 cases were predicted correctly by OCT, whereas for moderate dysplasia, the rate of correct identification was 32 of 38 cases. In the case of severe dysplasia, 24 out of 28 cases were correctly predicted, whereas for CIS, near-perfect agreement was observed in the form of 15 correctly classified out of 16 cases (93.8%).

**Figure 6:**
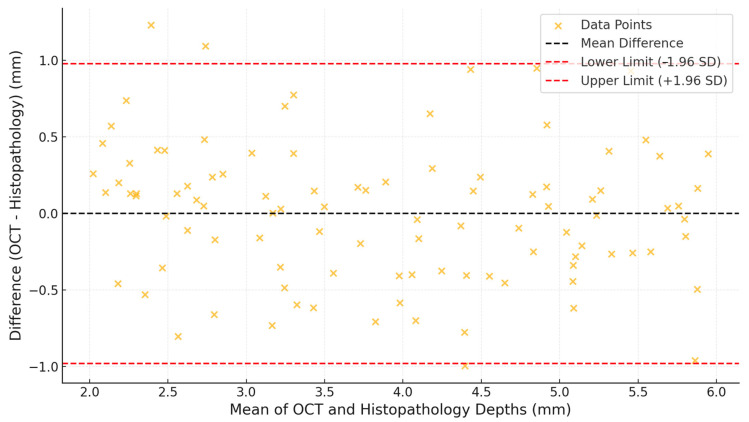
Bland–Altman plot of the agreement on tumour depth. This plot evaluates the agreement between OCT and histopathological measurements, showing the mean differences and 95% limits of agreement.

The agreement between the OCT and histopathological measurements is further highlighted in the Bland–Altman plot in Figure 6. The mean differences in the measurement accuracies of tumour depth were very small with tight limits of agreement. Hence, most data points fell inside the limits of agreement; this meant that there was less bias between OCT-derived measurements and histopathology-derived measurements. They clustered close to a zero-difference line, allowing high concordance from both methods even for deeper lesions.

### 3.6. Lesion Characteristics and Their Impacts on Diagnostic Performance

The diagnostic performance of OCT depended on the size of the lesion and its anatomical location. Lesions ≤ 5 mm exhibited higher sensitivity, 89.0%, and specificity, 91.0%, than larger lesions (>5 mm), which presented a sensitivity of 86.0% and a specificity of 89.0%, respectively (Table 6). This reflects the advantage of the resolution of OCT for smaller lesions with less complex architecture.

The results also showed significant differences concerning the diagnostic performance according to the lesion location. Thus, lesions located on the lateral tongue revealed a sensitivity of 90.0%, a specificity of 88.0%, and an AUC of 0.89, which were the highest values compared with those on the buccal mucosa, presenting an 85.0% sensitivity, 90.0% specificity, and 0.88 AUC. On the contrary, the keratinized OCT had higher sensitivity, amounting to 88.0%, while the specificity was lower and equalled 87.0%.

**Figure 7:**
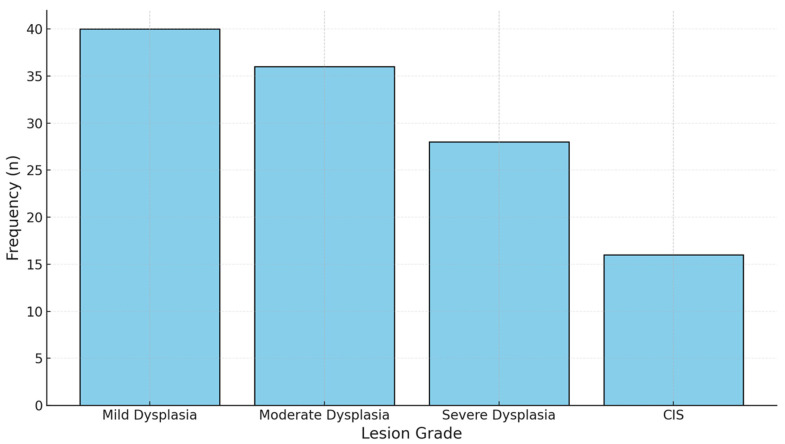
Stacked bar chart of the lesion grade distribution. This chart illustrates the frequency of lesions across histopathological grades (mild, moderate, severe dysplasia, and CIS), providing a clear visual distribution.

In Figure 7, a stacked bar plot representing the distribution of the histopathological grades within the lesions is shown; as presented in this graph, there were mostly mild and moderate cases, although severe dysplasias and CIS make up quite a substantial part of the lesions.

### 3.7. Margin Assessment Accuracy

The predictive capability of OCT for the margins of the lesion was compared with the histopathology results presented in Table 7. Mild dysplasia had an agreement rate of 91.6%, with the correct identification of thirty-five clear margins and five positive margins. In the case of moderate dysplasia, the agreement rate was 88.3%, where thirty-two clear margins and six positive margins were correctly identified. For severe dysplasia, the agreement rate was 85.7%, and for CIS, it was 93.3%.

Only a few false-positive and false-negative margin predictions were found; most of these were from severe dysplasia and CIS cases. For example, in one case of CIS, histopathologically confirmed positive margins were incorrectly classified as clear by OCT. Such findings indicate that while the accuracy of OCT in delineating lesion margins is high, it is not infallible.

**Figure 8:**
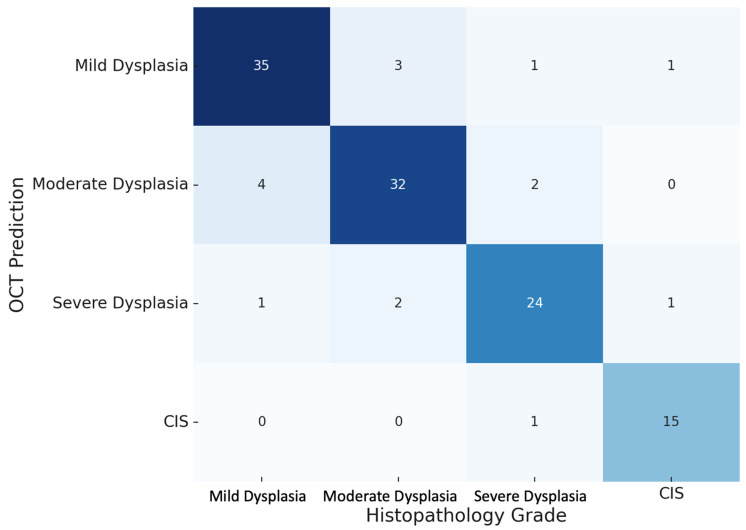
Heatmap of the OCT vs. histopathology agreement. This heatmap visualizes the agreement between the OCT predictions and histopathology-confirmed grades, highlighting strong correlations along the diagonal.

Margin predictions of OCT against dysplasia grades were further highlighted in the heat map presented in Figure 8. The figure strongly underlined a very sharp diagonal correlation between the OCT and histopathology predictions, hence indicating very strong agreements. Misclassifications were few and usually between adjacent grades of dysplasia. The diagonals that were underlined showed the high precision of OCT against the matching histopathological diagnosis, while the few prediction errors were spotted as off-diagonal data.

### 3.8. Predictive Imaging Features Identified by OCT

OCT imaging provided characteristic features for each grade of dysplasia and gave relevant diagnostic information. Details are given in Table 8. For mild dysplasia, the epithelium was thickened to 100–150 μm and basement membranes were intact, with low reflectivity. During moderate dysplasia, the epithelium became thickened to 150–200 μm, and the basement membranes were intact, while mixed reflectivity due to moderate architectural disorganization was obtained.

Severe dysplasia had an epithelial thickness of about 200–250 μm and basement membranes that were intact, while presenting high reflectivity patterns. The full development of changes was realized in CIS, where the epithelium was even thicker than 250 μm and accompanied by a disruption of the basal membrane with a severe disorganization of the architecture. Thus, subepithelial and especially stromal and vascular changes were very evident in CIS, reflecting its gross pathology.

**Figure 9:**
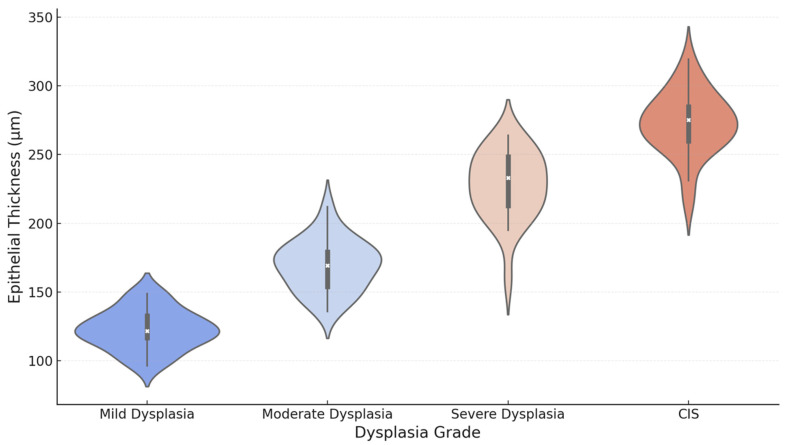
Violin plot of the epithelial thickness of each dysplasia grade. This violin plot shows the distribution of epithelial thickness across dysplasia grades, highlighting the trends and variability.

In Figure 9, a violin plot of the distribution of epithelial thickness among different dysplasia grades is shown. It can be seen that with the aggravation of dysplasia, the median epithelial thickness increased, and CIS had the largest variability and highest median thickness. The shape of the violin plot indicates the density of the measurement; the broader the violin, the higher the frequency.

### 3.9. Interobserver Agreement for OCT Interpretations

In total, interobserver agreement for the OCT interpretation showed overall substantial to almost perfect agreement for all dysplasia grades on the basis of Cohen’s kappa coefficient (Table 9). Correspondingly, for mild dysplasia, the κ value totalled 0.81. The values of the Cohen’s kappa statistic in regards to the presence of moderate and severe dysplasia were obtained as 0.77 and 0.74, while it was 0.85 for carcinoma in situ, respectively. Conclusively, such OCT results prove that consistency with trained observers may already allow for a consensus regarding most of the higher degrees of dysplasia.

### 3.10. Correlations Between the OCT Imaging Features and the Dysplasia Grade

Table 10 shows the statistical analysis of the correlation between OCT imaging features and histopathological grading. Epithelial thickness had the highest correlation coefficient (r = 0.82, <0.001), though it was closely followed by the reflectivity pattern and architectural disorganization (r = 0.78 and 0.79, respectively); basement membrane disruption presented a good positive correlation, contributing to its diagnostic validity.

There was a moderate positive correlation, with a value of r = 0.71, *p* < 0.001, between subepithelial changes, including hyporeflective areas and stromal involvement. These findings confirm the diagnostic value of OCT imaging features in differentiating dysplasia grades and highlight the potential for integrating quantitative metrics into routine OCT evaluations.

### 3.11. Integrated Diagnostic Insights

**Figure 10:**
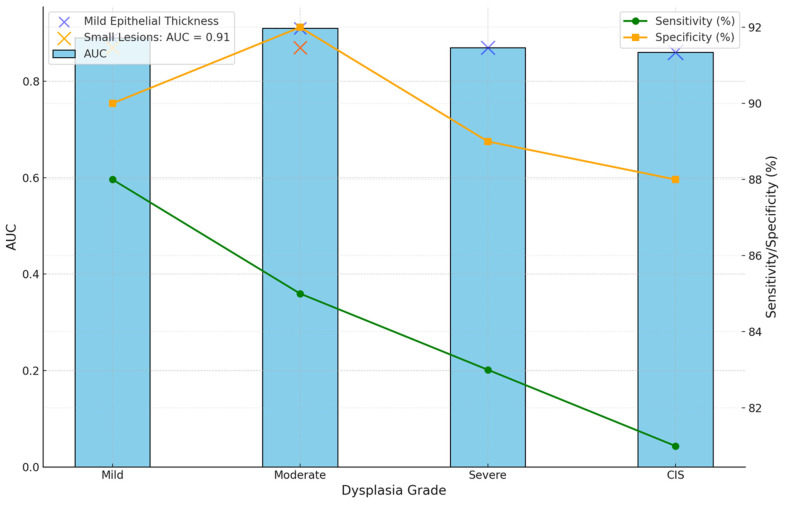
Integrated diagnostic insights into OCT performance and lesion features. This figure combines the AUC values, sensitivity, specificity, epithelial thickness, and lesion characteristics into a multi-dimensional visualization, providing a comprehensive overview of OCT’s diagnostic performance.

The overall diagnostic performance of OCT is summarized in the multi-dimensional visualization presented in Figure 10. This figure integrates the AUC values, sensitivity, specificity, epithelial thickness, and lesion characteristics into a cohesive representation that underlines the strong capabilities of OCT across diverse lesion profiles. The layered display of diagnostic metrics is unified in a view enabling clinicians to appreciate the role of OCT in tackling complex diagnostic challenges effectively.

## 4. Discussion

### 4.1. Challenges of the Study

This study evaluated the diagnostic performance of OCT for differentiating the grades of OED. Despite its robust methodology and promising findings, several challenges merit discussion. First, the study was conducted in a single-centre setting, which may limit generalizability to broader clinical populations with diverse lesion presentations and healthcare access. This inclusion of high-risk lesions enriched the data but, on the other hand, may have introduced selection bias in a way that might overestimate measures of diagnostic accuracy.

Another challenge is the interpretation of the OCT images. Interobserver agreement was high, and no doubt, training and expertise played an important role in that. How the findings would be replicated across operators with less experience remains uncertain. Also, there is a possibility that OCT images might be interpreted subjectively, and borderline dysplasia accounts for some degree of the misclassification rates observed.

Another weakness of the study is intrinsic: the limitations related to using histopathology as a gold standard. Biopsy samples just show a representative part of the lesion; there is always a concern of sampling error. In addition, despite the consensus review, there is still interobserver variability in histopathological grading that may be a possible confounding variable.

### 4.2. Discussion of the Results

The results from this study proved the efficiency of OCT as a diagnostic tool in assessing OED or CIS, its sensitivity for all grades of dysplasia, along with a good specificity above 80%. The corresponding ROC in this regard yielded an overall AUC of 0.91 for the classification of moderate dysplasias, pointing out the notably high diagnostic accuracy of OCT. This underlines the abilities of OCT to perform properly a non-invasive methodical evaluation of tissue architectonics, epithelial thickness, and basement membrane integrity and is of the highest importance for the proper detection of the markers peculiar to dysplasia.

The Bland–Altman analysis confirmed the concordance of OCT-derived tumour depth measurements with the gold standard of histopathology, with a minor bias observed. This firmed up the argument of OCT being able to assume the role of an invasive biopsy in selected cases. Also, the heatmap of OCT and histopathology agreement further provided substantial evidence for the utility of OCT for high-grade dysplasia and CIS.

Nevertheless, some of the limitations of the results included that while smaller lesions tended to result in higher diagnostic performance, larger lesions tended to be problematic, likely due to their heterogeneity and architectural complexity. Also, though keratinized lesions showed high sensitivity, their specificity was relatively lower, probably due to imaging artifacts or difficulties in interpretation.

Although the findings highlight OCT’s potential as a valuable diagnostic tool, its limitations must be acknowledged. OCT cannot provide the cellular-level detail essential for identifying certain histopathological features, such as subtle nuclear atypia or early invasion, which remain critical for a definitive diagnosis. Moreover, its reliance on operator expertise and the need for standardized imaging protocols can pose challenges to its routine adoption. While OCT offers significant potential as a non-invasive diagnostic modality, it must be emphasized that it is not a replacement for a histopathological analysis. OCT should instead be considered an adjunct tool that enhances clinical decision-making by aiding in lesion assessment and guiding the optimal site for biopsy. This integration ensures a comprehensive evaluation while reducing the risk of sampling error and improving the diagnostic accuracy.

### 4.3. Discussion of the Results in Relation to Relevant Studies

The diagnostic performance of OCT, as evident in this study, agrees fairly well with most of the literature and tends to bring out the increasing importance of OCT as an asset in oral pathology, medicine and surgery. Sensitivities ranging between 88% and 91% for the grades of dysplasia somewhat compare with a sensitivity of 91% and a specificity of 93% achieved in the study undertaken by Wilder-Smith et al. in order to diagnose oral dysplasia and malignancy [9]. Such a finding shows that OCT gives robust tissue assessment results under various non-invasive scenarios.

Tsai et al. applied swept-source OCT for the detection of early markers of oral cancer and reported 75% sensitivity, which is less than that obtained in our series; it still showed that the technique is effective for detecting microstructural changes [12]. Lee et al. also applied OCT for discriminating between normal and dysplastic tissues, demonstrating its effectiveness by providing a sensitivity of 82% and specificity of 90%. All of those values closely parallel our observations, in particular, the trend of discriminating moderate dysplasia; this widely reinforces OCT’s reliability independent of the lesion type [13].

Hamdoon et al. [14] further corroborated the value of OCT in assessing the surgical margins of OSCC by reporting metrics of diagnostic performance similar to that of our margin delineation study. They also identified the potential of OCT in reducing incomplete resections and recurrence rates through its utilities in surgical planning and as a real-time guide in surgery.

The integration of artificial intelligence with OCT has taken things to the next level in offering diagnostic precision. Yuan et al. reported that this approach, with the assistance of AI-enhanced OCT, realized a remarkable sensitivity and specificity of 98% and 99%, respectively [15]. Thus, AI enhancement significantly improved outcomes for malignant changes. Comparable studies by Sunny et al. further demonstrated perfect diagnostic metrics with sensitivity and specificity values of 100%, each derived using AI-driven OCT and thus delineating tumour margins. These developments complement our work on the margin assessment and overcome important limitations, including interobserver variability and misclassification rates [16].

Yang et al. extended these features by incorporating a texture analysis into OCT imaging. They have reported diagnostic performance metrics for their approach comparable to those of AI-driven methods. Their findings are in conjunction with our findings of the epithelial thickness and basement membrane disruption as key markers of dysplasia and malignancy, underlining the usefulness of OCT in detecting architectural features that are subtle and not easily visible [17].

Obade et al. confirmed the role of OCT in differentiating precancerous lesions based on assessing epithelial thickness and vascular changes in the subepithelium, which are also two key features in our study. The results confirm that OCT plays a significant role in the early detection of malignant changes and further underlines its value for diagnostics and prognosis [18].

Finally, the present study aligns with the broader advancements in OCT technology, such as those demonstrated by Chang et al., who developed birefringent tissue-mimicking phantoms for polarization-sensitive OCT imaging. Their innovative approach highlights the potential of polarization-sensitive OCT to provide additional diagnostic insights by assessing the structural and birefringent properties of tissues, such as collagen organization. While our study focused on conventional OCT for detecting and grading oral epithelial dysplasia, the incorporation of polarization-sensitive techniques could further enhance the diagnostic accuracy by enabling an evaluation of stromal remodelling, which often accompanies dysplasia and carcinoma. This underscores the evolving role of OCT in oral diagnostics, suggesting that future iterations of the technology could integrate advanced imaging modalities to capture more comprehensive tissue characteristics, ultimately improving clinical outcomes [19].

Collectively, these studies reinforce the very high diagnostic accuracy and wide range of clinical utilities provided by OCT. Variability in the sensitivities and specificities from heterogeneity within study designs and different OCT systems are apparent, yet the general consensus reinforces that OCT will be an important intervention in diagnosing and managing oral dysplasia. Advanced technologies such as AI and texture analysis improve the already considerable potential of OCT in new ways of improving the diagnosis and patient outcomes.

### 4.4. Clinical Implications

For the clinician, OCT may prove a useful adjunct for the early detection and monitoring of OED, especially in high-risk patients. Grading dysplasia will influence treatment planning, with the potential to tailor the intervention according to the lesion severity. High accuracy in margin delineation also supports the use of OCT in surgical planning for the prevention of incomplete excisions and recurrences.

In resource-poor environments, OCT offers great portability and ease of use compared to most diagnostic methods. The ability to perform in vivo imaging without a special infrastructure may extend early detection and improve outcomes in underserved populations.

### 4.5. Future Directions

In general, the results of the study throw open several lines of prospective studies. The need for future studies to include a large number of patients from multiple centres with varied populations would further establish the general validity of such results. Also, for review purposes, there should have been more varied types and anatomical sites of the lesion for comprehensive evaluations of the diagnostic performance of OCT.

This could be facilitated by an artificial intelligence-based OCT interpretation, the future of which is deemed very promising. Machine-learning algorithms could lead to an accurate diagnosis, reducing the interobserver variability in the automated classification of lesions. Further discussion is warranted regarding the feasibility in routine clinical practice in studies to come.

Standardization of the protocols, including imaging parameters and interpretation criteria, is very important in OCT studies for consistency among various studies and clinical settings. The development of guidelines through a collaborative effort will facilitate wider acceptance and enhance diagnostic reliability.

Finally, studies comparing OCT to other non-invasive modalities, such as CLE (Confocal laser endomicroscopy) and narrowband imaging, would be instructive. Assessing the value of various imaging techniques in concert might further expand its diagnostic potential.

## 5. Conclusions

The current study demonstrated the high accuracy for OCT in the differentiation of the grades of OED and thereby established this technology as a possible non-invasive, real-time diagnostic modality. Thus, the present work, which correlates the results with the histopathological appearance, studies the reliability of the assessment of the lesion architecture, epithelial thickness, and margin status.

Although OCT has limitations in the interpretation of complex lesions and variability among operators, it is a significant development in early detection and management. Full integration into clinical practice could enhance the precision of lesion assessments and improve patient outcomes by enabling clinicians to select optimal biopsy sites. However, OCT is not a replacement for biopsy and must be used in conjunction with a histopathological analysis to ensure an accurate diagnosis and management, particularly for high-risk or ambiguous lesions.

Future studies should be directed toward overcoming the identified challenges by capitalizing on technological advances and standardizing protocols to achieve the maximum utilization of OCT. With continued innovation and validation, OCT has the potential to revolutionize the diagnosis of oral precancer and cancer.

## Figures and Tables

**Table 1:** Patient and sample characteristics.

Characteristic	Value	Characteristic	Value
Number of Patients	120	Number of Lesions	120
Age (years), mean ± SD *	56.8 ± 11.7	Age Range (years)	29–81
Gender (male/female)	67/53	Family History of Oral Cancer (Yes/No)	14/106
Smoking Status (current/former/never)	46/26/48	Alcohol Consumption (Yes/No)	68/52
Heavy Alcohol Use (Yes/No)	19/101	Betel Quid Use (habitual/non-users)	12/108
Chronic Irritation (Yes/No)	32/88	Poor Oral Hygiene (Yes/No)	52/68
Comorbidities (Yes/No)	24/96	Vitamin Deficiency (Yes/No)	16/104
History of Immunosuppression (Yes/No)	8/112	Use of Photosensitizing Drugs (Yes/No)	9/111
Oral HPV Suspected (Yes/No)	12/108	HPV Vaccination (Yes/No)	15/105

* SD: standard deviation.

**Table 2:** Clinical characteristics of lesions.

Characteristic	Value	Characteristic	Value
Lesion Size (mm), mean ± SD *	7.2 ± 1.8	Lesion Size Range (mm)	4–10
Lesion Duration (weeks), mean ± SD *	6.3 ± 2.7	Lesion Duration Range (weeks)	3–12
Lesion Symptomatology (Symptomatic/Asymptomatic)	72/48	Lesion Ulceration (Yes/No)	36/84
Lesion Appearance (Keratinized/Non-Keratinized)	58/62	Lesion Type: Leucoplakia (Homogeneous)	50 (42%)
Leucoplakia (Non-Homogeneous)	28 (23%)	Lesion Type: Erythroplakia	28 (23%)
Mixed Red and White Lesions	14 (12%)	Lesion Location: Lateral Tongue	42 (35%)
Lesion Location: Buccal Mucosa	28 (23%)	Lesion Location: Floor of the Mouth	26 (22%)
Lesion Location: Soft Palate	14 (12%)	Lesion Location: Retromolar Trigone	10 (8%)

* SD: standard deviation.

**Table 3:** Histopathological grading of lesions.

Lesion Grade	Frequency (n)	Percentage (%)
Mild Dysplasia	40	33.3
Moderate Dysplasia	36	30.0
Severe Dysplasia	28	23.3
Carcinoma In Situ (CIS)	16	13.3

**Table 4:** Diagnostic accuracy metrics for OCT.

Lesion Grade	Sensitivity (%)	Specificity (%)	PPV * (%)	NPV * (%)	AUC *
Mild Dysplasia	88.0	90.0	85.7	91.7	0.89
Moderate Dysplasia	85.0	92.0	86.7	90.2	0.91
Severe Dysplasia	83.0	89.0	82.1	90.0	0.87
Carcinoma In Situ (CIS)	81.0	88.0	80.2	89.5	0.86

* PPV: positive predictive value; NPV: negative predictive value; AUC: area under the curve.

**Table 5:** OCT vs. histopathology agreement.

Histopathology Grade	Mild Dysplasia	Moderate Dysplasia	Severe Dysplasia	CIS	Total
OCT Prediction					
Mild Dysplasia	35	3	1	1	40
Moderate Dysplasia	4	32	2	0	38
Severe Dysplasia	1	2	24	1	28
Carcinoma in Situ (CIS)	0	0	1	15	16
Total	40	37	28	17	122

**Table 6:** Lesion characteristics and diagnostic performance.

Lesion Characteristic	Sensitivity (%)	Specificity (%)	AUC *
Small Lesions (≤5 mm)	89.0	91.0	0.90
Large Lesions (>5 mm)	86.0	89.0	0.88
Lateral Tongue	90.0	88.0	0.89
Buccal Mucosa	85.0	90.0	0.88
Keratinized Lesions	88.0	87.0	0.87
Non-Keratinized Lesions	84.0	89.0	0.86

* AUC: area under the curve.

**Table 7:** Margin assessment accuracy.

Lesion Grade	OCT Prediction (Clear/Positive)	Histopathology (Clear/Positive)	Agreement (%)
Mild Dysplasia	35/5	34/6	91.6
Moderate Dysplasia	32/6	30/8	88.3
Severe Dysplasia	24/4	23/5	85.7
Carcinoma In Situ (CIS)	15/1	14/2	93.3

**Table 8:** Predictive imaging features identified by OCT for dysplasia grading.

Imaging Feature	Mild Dysplasia	Moderate Dysplasia	Severe Dysplasia	CIS
Epithelial Thickness (µm)	100–150	150–200	200–250	>250
Reflectivity Patterns	Low	Mixed	High	High
Architectural Disorganization	Absent or mild	Mild to moderate	Moderate to severe	Severe
Basement Membrane Integrity	Intact	Intact	Intact	Disrupted
Subepithelial Changes	None	Hyporeflective areas	Stromal involvement	Stromal and vascular changes

**Table 9:** Interobserver agreement for OCT interpretations.

Lesion Grade	Observer 1 Prediction (n)	Observer 2 Prediction (n)	Cohen’s Kappa (κ)	Interpretation of Agreement
Mild Dysplasia	38	37	0.81	Almost Perfect
Moderate Dysplasia	36	35	0.77	Substantial
Severe Dysplasia	27	28	0.74	Substantial
Carcinoma In Situ (CIS)	15	16	0.85	Almost Perfect

**Table 10:** Correlations between OCT imaging features and the dysplasia grade.

Imaging Feature	Pearson’s Correlation Coefficient (r)	*p*-Value	Significance
Epithelial Thickness	0.82	<0.001	Strong positive correlation
Reflectivity Pattern	0.78	<0.001	Strong positive correlation
Basement Membrane Disruption	0.75	<0.001	Strong positive correlation
Architectural Disorganization	0.79	<0.001	Strong positive correlation
Subepithelial Changes	0.71	<0.001	Moderate positive correlation

## Data Availability

The data that support the findings of this study are available upon request from the corresponding author (W.J.).
